# Biopsy and Margins Optimize Outcomes after Thermal Ablation of Colorectal Liver Metastases

**DOI:** 10.3390/cancers14030693

**Published:** 2022-01-29

**Authors:** Nikiforos Vasiniotis Kamarinos, Efsevia Vakiani, Mithat Gonen, Nancy E. Kemeny, Carlie Sigel, Leonard B. Saltz, Karen T. Brown, Anne M. Covey, Joseph P. Erinjeri, Lynn A. Brody, Etay Ziv, Hooman Yarmohammadi, Henry Kunin, Afsar Barlas, Elena N. Petre, Peter T. Kingham, Michael I. D’Angelica, Katia Manova-Todorova, Stephen B. Solomon, Constantinos T. Sofocleous

**Affiliations:** 1 Interventional Oncology/IR Service, Department of Radiology, Memorial Sloan Kettering Cancer Center, New York, NY 10065, USA; nikiforosvasikama@gmail.com (N.V.K.); ktbirmd@gmail.com (K.T.B.); coveya@mskcc.org (A.M.C.); erinjerj@mskcc.org (J.P.E.); brodyl@mskcc.org (L.A.B.); zive@mskcc.org (E.Z.); yarmohah@mskcc.org (H.Y.); kuninh@mskcc.org (H.K.); petree@mskcc.org (E.N.P.); solomons@mskcc.org (S.B.S.); 2 Department of Pathology, Memorial Sloan Kettering Cancer Center, New York, NY 10065, USA; vakianie@mskcc.org (E.V.); sigelc@mskcc.org (C.S.); 3 Department of Epidemiology & Biostatistics, Memorial Sloan Kettering Cancer Center, New York, NY 10065, USA; gonenm@mskcc.org; 4 Department of Medicine, Memorial Sloan Kettering Cancer Center, New York, NY 10065, USA; kemenyn@mskcc.org (N.E.K.); saltzl@mskcc.org (L.B.S.); 5 Molecular Cytology, Memorial Sloan Kettering Cancer Center, New York, NY 10065, USA; barlasa@mskcc.org (A.B.); manovak@mskcc.org (K.M.-T.); 6 Department of Surgery, Memorial Sloan Kettering Cancer Center, New York, NY 10065, USA; kinghamP@mskcc.org (P.T.K.); dangelim@mskcc.org (M.I.D.)

**Keywords:** thermal ablation, colorectal liver metastases, post-ablation biopsy, ablation margin assessment

## Abstract

**Simple Summary:**

Thermal ablation (TA) is a non-surgical treatment of cancer that has been used with success in the treatment of colorectal liver metastases (CLM). TA consists of burning the cancer and a rim of surrounding tissue (margin) with a special needle placed in the tumor under image guidance. Despite the technological evolution of TA, tumor progression/recurrence rates remain higher than expected. We present a method that combines tissue and imaging tests performed immediately after ablation to determine whether there is complete tumor destruction or remaining live cancer cells that can cause tumor progression/recurrence. This information can provide guidance for additional treatments for patients with evidence of residual cancer, i.e.,: additional TA at the same or subsequent sitting, or additional chemotherapy and short-interval imaging follow-up to detect recurrence. The presented method proposes a clinical practice paradigm change that can improve clinical outcomes in a large population of patients with CLM treated by TA.

**Abstract:**

Background: Thermal ablation is a definitive local treatment for selected colorectal liver metastases (CLM) that can be ablated with adequate margins. A critical limitation has been local tumor progression (LTP). Methods: This prospective, single-group, phase 2 study enrolled patients with CLM < 5 cm in maximum diameter, at a tertiary cancer center between November 2009 and February 2019. Biopsy of the ablation zone center and margin was performed immediately after ablation. Viable tumor in tissue biopsy and ablation margins < 5 mm were assessed as predictors of 12-month LTP. Results: We enrolled 107 patients with 182 CLMs. Mean tumor size was 2.0 (range, 0.6–4.6) cm. Microwave ablation was used in 51% and radiofrequency ablation in 49% of tumors. The 12- and 24-month cumulative incidence of LTP was 22% (95% confidence interval [CI]: 17, 29) and 29% (95% CI: 23, 36), respectively. LTP at 12 months was 7% (95% CI: 3, 14) for the biopsy tumor-negative ablation zone with margins ≥ 5 mm vs. 63% (95% CI: 35, 85) for the biopsy-positive ablation zone with margins < 5 mm (*p* < 0.001). Conclusions: Biopsy-proven complete tumor ablation with margins of at least 5 mm achieves optimal local tumor control for CLM, regardless of the ablation modality used.

## 1. Introduction

Thermal ablation (TA) techniques, including radiofrequency (RFA) and microwave (MWA) ablation, use cytotoxic levels of thermal energy to destroy cancer cells in situ [1,2]. This can provide local control for selected colorectal liver metastases (CLM) with minimal risk [3,4,5,6]. TA has been incorporated into oncologic guidelines as a stand-alone therapy or in combination with surgery, as long as all visible disease can be eradicated [3]. Nevertheless, a residual viable tumor may not always be detected given the presently available morphologic and metabolic imaging methods, and this can lead to local tumor progression (LTP) [1].

Achieving ablation margins greater than 5 mm is considered to be the most important technical factor for local tumor control after TA [6,7,8,9,10,11,12]. Traditionally, the minimal ablation margin has been evaluated using anatomic imaging [8]. Despite improved accuracy of 3D measurements, all currently available methods of ablation margin assessment face several limitations [8,13].

Similar to the morphologic evaluation of frozen sections during surgical excision, prior studies have proposed the pathologic examination of tissue from the center and margin of the ablation zone (AZ) as seen in dynamic CT immediately following the ablative treatment of CLM [14]. This study presents 10-year experience using this method and evaluates the role of immediate post-ablation biopsy as an independent predictor of LTP regardless of TA modality.

## 2. Materials and Methods

This is a prospective, single-group, phase II study, conducted in a tertiary cancer center in the US (NCT01494324). It evaluated the anti-tumor activity of TA for CLM by comparing local control outcomes stratified by ablation margins and post-ablation biopsy results. No control group was required for this study [15].

### 2.1. Eligibility Criteria and Treatment

Patients undergoing TA ablation of CLM were assessed for enrollment in this HIPPA-compliant institutional review board-approved prospective study. Eligible patients had up to three CLM (each <5 cm in largest diameter) and no more than three, stable/controlled or treated extrahepatic sites of disease (including lymph nodes and pulmonary nodules).

All procedures were performed under general anesthesia with continuous hemodynamic monitoring by an anesthesiologist. The choice of the ablation system depended on availability, operator preference, and tumor size, shape, and location. In all cases, the manufacturer-recommended protocol for the desired specific size of each ablation was completed. Overlapping ablations were performed in order to achieve the desired configuration of the ablation zones completely covering the target tumor(s) with margins of at least 5 mm all around [1]. This endpoint was defined as a technical success and confirmed with a triphasic CT immediately after ablation in all cases [1].

### 2.2. Margin Assessment

The ablation margins were measured using previously described 2D manual [8] and 3D software-assisted [13] methods. In the manual method, the distance between the tumor edge and nearest reliable landmarks in different directions is measured on the preablation CT and then, the distance between the same landmarks and the edge of ablation differences on the post-ablation CT. The margin at each landmark is obtained by subtracting the pre-ablation distance from the post-ablation distance. The smallest value is considered the minimal margin (MM). In the 3D method, the minimal margin is determined by automatically computing the volume of coverage after semi-automated 3D registration and segmentation of the tumor and ablation zone. Minimal margin was assessed intra-procedurally and used to direct the post-ablation biopsy and reassessed on the first post-ablation liver triphasic contrast-enhanced CT within 4–8 weeks after ablation for the purpose of being evaluated as a factor affecting LTP, consistent with prior methodology [8,13].

### 2.3. Histopathologic Analysis

Immediately after ablation core needle biopsies were obtained from the center (where the tumor used to be) and the minimal margin of the ablation zone (AZ). As a minimum requirement, at least one core biopsy had to be obtained from the center of the AZ. A total of 352 samples (average 1.9 samples per AZ) were acquired: 182 from the ablation zone center; 12/182 samples included the center and the minimal margin (MM) of the AZ and 170/352 samples were obtained from the MM. Tissue fragments adherent to the RF or the MW applicators were also collected whenever present. Morphologic evaluation using hematoxylin-eosin (H&E) classified specimens as necrotic, if they displayed only necrosis/thermal artifact and did not contain tumor cells. Specimens that contained any tumor cells at H&E were further evaluated with immunohistochemistry (IHC) for proliferative activity (Ki-67), mitochondrial viability (OxPhos antibody, OXP), and apoptosis (Caspase-3) [16,17]. Specimens that were positive for Ki-67 and/or OXP antibodies were classified as viable tumors (VT), while specimens negative for Ki-67 and OXP and positive for caspase-3 were classified as necrotic.

### 2.4. Imaging Follow-Up

To evaluate thermal ablation efficacy and confirm the minimal ablation margin (MM) size, liver triphasic contrast-enhanced CT (CECT) was performed within 4–8 weeks after ablation [1]. This first post-ablation imaging was considered the new baseline for further comparisons according to reporting standards for ablation [1]. Further imaging was performed at 2–4-month intervals for up to 3 years after ablation and was prospectively assessed by study-dedicated faculty. Evidence of tumor progression within 1cm from the AZ seen on CT was considered LTP [1]. Patients with evidence of LTP were re-treated if they were still eligible for inclusion in the study.

### 2.5. Definitions

Thermal ablation efficacy: ablation zone (AZ) completely covering the target tumor with absence of enhancement within the ablated area in the first 4–8-weeks post-ablation CT scan [1].

Time-to-LTP: time-to-LTP was defined as the time between ablation and the first radiological evidence of LTP. This definition is made separately on the basis of each ablated tumor and, as a result, this is a tumor-specific endpoint.

Overall survival (OS): time between initial TA and patient death or most recent follow-up [1]. OS is a patient-based outcome.

Central biopsy: biopsy performed in the area of the AZ where the tumor previously resided.

Marginal biopsy: biopsy sampling the presumed minimal margin of the AZ on the immediate post-ablation CT.

Complications: any complications within 30 days of TA [1]. Complications that resulted in increased level of care and required hospitalization were considered major complications. All other complications were considered minor.

### 2.6. Statistical Analysis

The primary endpoint was to establish that viable tumor identified in tissue biopsy from the ablation zone and ablation margins < 5 mm are independent predictors of 12-month local tumor progression. The secondary aim was to evaluate whether LTP of ablated CLM is associated with overall survival. Median follow-up time was determined using the median follow-up time among the living patients at study completion. Each ablated tumor was considered an independent event. For OS analysis, only the first ablated tumor per patient was included. Multivariate analysis was performed by including the variables with a *p*-value < 0.05 at univariate analysis. To account for competing events of death or loss to follow-up before evidence of LTP (69/178—39% of tumors), a competing-risks regression model was used to assess time-to-LTP. [18]. The HR of LTP for the subset of patients with margins < 5 mm and positive biopsy (in reference to the group with ≥5 mm margins and negative biopsy) was estimated because this regression model is additive in log hazards [18]. The effect of LTP on OS was estimated using a time-varying covariate. Stata 12 software (Stata, College Station, TX, USA) was used for statistical analysis.

## 3. Results

**Figure 1:**
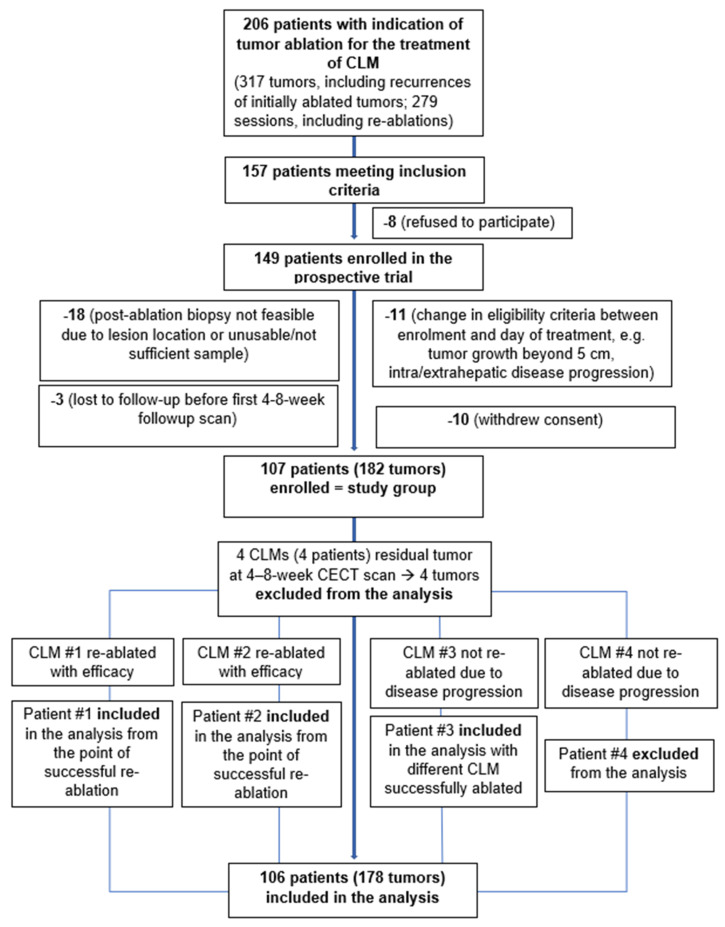
Application of inclusion/exclusion criteria for determining the study group.

Between November 2009 and February 2019, 107 consecutive patients (65 men, 42 women; age range, 32–82 years) with 182 CLM treated with image-guided TA, met the eligibility and were included in this prospective study. RFA was used in 90 (49%) and MWA in 92 CLMs (51%). Table 1 and Table S1, Figure 1 displays a step-by-step application of inclusion/exclusion criteria arriving at the study population.

### 3.1. Thermal Ablation Efficacy

Efficacy was achieved in 178/182 (98%) ablated tumors. In four tumors (four individual patients), there was evidence of residual unablated tumor in the first CT scan 4–8 weeks after ablation. All four tumors had a tumor-positive post-ablation biopsy. Two/four of these tumors were re-ablated within the study, and efficacy was achieved. In the other two patients, the first post-ablation CT scan also demonstrated evidence of progression of disease in the retroperitoneum and the lymph nodes; these patients received chemotherapy without any further ablation. Thus, the four tumors were excluded from the analysis of the primary aim (LTP). The two patients who had their tumors successfully re-ablated were included in the analysis of patient-based outcomes, accounting from the point of technical efficacy. The third patient had another tumor that was ablated successfully, and was included in the analysis for that tumor. The final patient was excluded from the analysis. Tumors/patients exclusions are reflected in the study group diagram (Figure 1).

### 3.2. Tissue Findings

H&E staining identified tumor cells in 89 biopsy specimens (55 from the center, 29 from margin biopsies, and 5 from the electrodes) from 64 CLM.

To evaluate the detected tumor cell viability and proliferation potential with IHC, the study pathologist chose the most representative sample containing tumor cells from each AZ (*n* = 64). Fifty-five/64 (86%) specimens were Ki-67 and OXP positive and one was Ki-67 negative, OXP positive, and caspase-3 negative; this specimen was classified as viable tumor (VT). In 8/64 ablation zones in which H&E detected intact tumor cells, tissue amount was insufficient for IHC evaluation. These samples were classified as VT. In summary, 64 tumors were classified by post-ablation biopsy as VT and 114 as necrotic.

Specimens from the AZ center were more likely to contain tumor cells than specimens from the margin (*p* = 0.004). There was no association between margin size and biopsy result. Specifically, 48/145 (33%) AZ with minimal margin size ≥ 5 mm and 16/33 (48%) AZ with a margin size < 5 mm contained tumor cells in the corresponding biopsy samples (*p* = 0.073).

### 3.3. LTP Findings

Cumulative incidence of LTP at 12 and 24 months was 22% (95% confidence interval [CI]: 17, 29) and 29% (95% CI: 23, 36) respectively. Within the 31 months of study median follow-up, LTP occurred in 33/64 (52%) tumors classified by post-ablation biopsy as viable and in 25/114 (22%) tumors classified as necrotic (*p* < 0.001).

Tumor size, minimal margin size, biopsy result, and prior liver resection were significant predictors of time-to-LTP at univariate analysis (Table 2).

Positive post-ablation biopsy (hazard ratio [HR], 2.4; *p* = 0.002) and minimal ablation margin size (<5 mm) (HR, 3.5; *p* < 0.001) were independent predictors of shorter time-to-LTP (Table 3).

**Figure 2:**
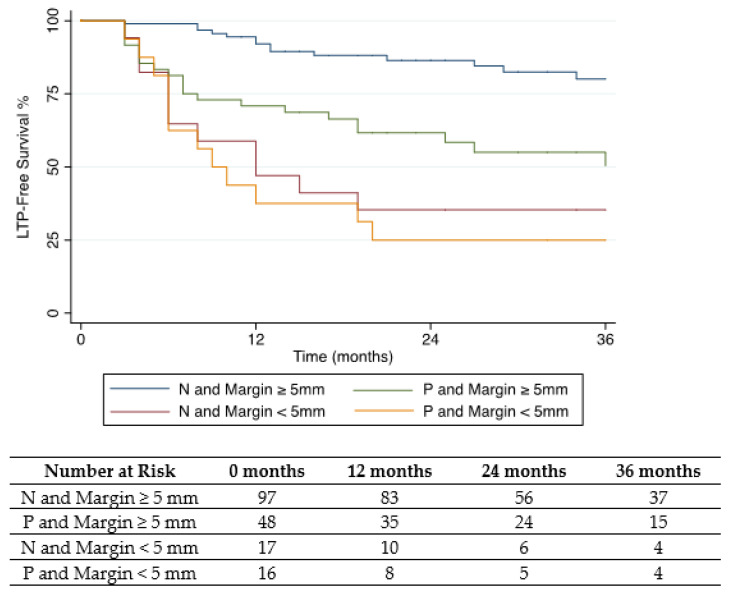
Kaplan–Meier curve of local tumor progression free survival stratified by post-ablation biopsy result (N: Negative or P: Positive) and ablation margin size.

**Figure 3:**
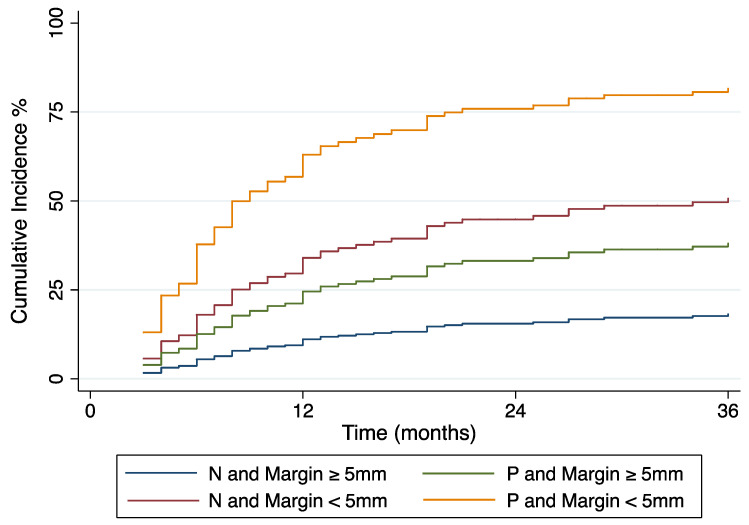
Cumulative incidence of local tumor progression over time stratified by post-ablation biopsy result (N: Negative or P: Positive) and ablation margin size.

The HR of LTP for a tumor with narrow margins and positive tissue biopsy was 20.3 (95% CI: 4.5, 48.2). LTP within the first 12 months after TA occurred in seven/97 (7%; 95% CI: 3, 14) of biopsy-negative ablation zones with minimal margins ≥ 5 mm, and 10/16 (63%; 95% CI: 35, 85) biopsy-positive ablation zones with margins < 5 mm (*p* < 0.001). Cumulative incidence and Kaplan–Meier survival curves are displayed in Figure 2 and Figure 3.

### 3.4. Patient Survival

Overall survival rate from the date of ablation was 92% (95% CI: 85, 96) at 12 months, 73% (95% CI: 64, 81) at 24 months, 56% (95% CI: 46, 65) at 36 months, and 35% (95% CI: 25, 45) at 5 years. The median OS from the date of ablation and from the date of initial diagnosis of colorectal cancer was 46 months (95% CI: 34, 54), and 84 months (95% CI: 77, 96), respectively. The HR of LTP to OS was 1.4 (95% CI: 0.9, 2.3; *p* = 0.13). Pre-ablation CEA levels >30 ng/mL (*p* < 0.001), and systemic chemotherapy post-ablation were associated with reduced OS (*p* < 0.001). Patient characteristics as predictors of OS from ablation date are displayed in Table 4.

### 3.5. Complications

Four instances of pneumothorax were recorded, all treated with thoracostomy without further sequelae, and were classified as minor complications. Major complications occurred in four patients. The first patient presented with fever, chills, and right upper quadrant pain after ablation of a recurrent CLM near the surgical margin. A large biloma that was present in the area of surgical resection before ablation developed locules of air detected in post-ablation CT representing infection. The collection was drained, and the patient recovered after receiving a course of antibiotics. The second patient complained of right upper quadrant pain 2 weeks after ablation near a prior (resolved) post-hepatectomy abscess. Imaging 3 weeks post-ablation showed evidence of bleeding and abscess recurrence with bile leak. The patient was treated with embolization and biliary drainage without further sequelae. Another patient developed fever and thrombocytopenia 45 days after ablation. An asymptomatic intrahepatic hematoma caused by a right hepatic artery pseudoaneurysm was detected on the post-ablation CT with contrast. This resolved after embolization without further sequelae. The fourth complication was a case of pulmonary embolism occurring 2 weeks after ablation of two CLM in the same session and resolved with anticoagulation treatment.

## 4. Discussion

This study demonstrates that margins and biopsy of the AZ are independent predictors of LTP. Contrary to prior reports, in this cohort neither tumor size nor ablation modality was a predictor of LTP [6,19,20]. The concept of tumor-free margins is applied in locoregional therapies, including surgical resection, radiotherapy, and image-guided percutaneous ablation [6,21,22,23,24]. The presence of micrometastases not detected by imaging, adjacent to the target CLM is responsible for recurrent tumor growth even after complete local tumor treatments [25]. A significant difference between TA and surgical excision of CLM is the lack of pathological evidence of complete tumor eradication with negative margins [24,25]. The morphologic examination of the surgical margins for cancer cells is a process inherent to surgical excision, whereas it has not been applied in image-guided therapies such as TA and radiotherapy. Prior studies indicated that positive surgical margins are associated with higher risk of tumor recurrence and shorter survival [24,26]. TA eliminates cancer cells in situ by applying heat into the target tumor. The outcome of TA is evaluated by measuring the post-ablation margins in imaging [8,13]. An adequate margin is the most important technical factor contributing to local tumor control [6,7,8,9,22,27,28]. The results of the present study prospectively confirm that a circumferential 5 mm margin around the target CLM is the minimal requirement to achieve local tumor control [29]. This circumferential margin measured relying only on the conventional 2D, side-by-side, landmark-based approach described in earlier studies is limited [8]. The 2D assessment is time-consuming, and most importantly, cannot reliably discriminate between tumors that will eventually progress versus those that will not [13]. Several methods have been described regarding the assessment of the ablation zone after TA of liver tumors [30,31,32,33,34,35,36,37,38,39,40,41]. Assessing minimal margin in all anatomical planes is desirable and can be accomplished by the use of 3D models [13,34,36,37,38]. It has been shown that multiplanar, stereotactic volumetric assessments can provide more reliable measurements than the 2D method [13,40,42,43], that is prone to error independently of the interventional radiologist’s expertise in percutaneous tumor ablation [43]. However, both 2D and 3D methods rely on imaging. Despite progress in image fusion and registration software [37], accurate measurement of the ablation margin may still be challenging, especially when using images from scans performed at different times. Moreover, technical factors such as patient or table movement during ablation or organ movement after hydro or air-dissection to protect vital anatomic structures may increase further the error in ablation margin measurement. In the absence of these factors, radiologic-pathologic correlation in resected specimens has shown that the radiographic AZ lies within 2 mm of the histopathologic ablation zone [44]. The critical 1–2 mm distance difference that can turn adequate ablation margins to suboptimal or vice versa can be easily miscalculated with currently available imaging techniques.

In an effort to explain the incidence of LTP after a radiographically successful ablation, prior investigators examined tissue from RF electrodes and showed that the presence of tumor cells after treatment was correlated with oncologic outcomes [45,46]. A prior prospective study found residual viable and prolific tumor cells in biopsies immediately after RF ablation of CLM [14]. The ability of these residual cancer cells to proliferate, as shown by Ki67 positivity, may reflect resistance to heating or an increased activity after suboptimal hyperthermic heating that leads to LTP [17,47]. The present study shows that the immediate post-ablation biopsy of the AZ is associated with LTP when performed alone (*p* < 0.001) or in combination with margin assessment (HR 2.4, 95% CI: 1.4,4.1) regardless of the use of RF or MW as the source of TA. Due to the challenges associated with ablation margin assessment even with dedicated 3D software applications, we recommend including the biopsy whenever possible, especially when the tumor cannot be ablated with optimal margins.

The 12-month rate of LTP for a tumor-negative AZ biopsy with margins of at least 5 mm was 7% (95% CI: 3, 14). This rate is comparable to the 0–5% LTP rate reported [22,48,49] for ablation margins >10 mm and introduces an alternative ablation treatment option for tumors that cannot be ablated with wide margins due to their anatomical location and vicinity to critical structures [6] as well as those at risk for post-ablation biliary complications [48].

Although biopsy was associated with a lower HR compared to the ablation margin in the multivariate analysis, it introduces the most objective tool for ablation effectiveness evaluation [44]. Tissue assessment is less vulnerable to operator variability [50] and technical limitations, such as those described previously with regards to measurement of the ablation margin by imaging [50,51]. We also demonstrated that the pathologic evidence of tumor cells is not associated with the size of the ablation margin (*p* = 0.073). The latter finding highlights the value of immediate post-ablation biopsy not only in cases where minimal margin measurements are challenging and less accurate but also in cases where margin evaluation methods perform well. The H&E classification proved to be highly concordant with the IHC for biopsy specimens that were positive for tumors in this cohort. Post-ablation identification of tumor cells has been used for morphological diagnostic purposes [52]; however, even in the eyes of experienced pathologists it is impossible to classify necrotic vs. viable tumor cells by morphology only. This is the purpose of adding IHC as described in this work. IHC identifies viable (OXPHOS AB) and prolific (Ki67) tumor cells that eventually lead to LTP after ablation [14,17,53].

A key limitation of the post-ablation biopsy is that specimens from the AZ center and margin may not reflect tumor necrosis or viability within the entire ablated tissue volume as opposed to the excised surgical specimens. In addition, the initially presumed minimal margin, estimated by CT immediately post-ablation and targeted with a biopsy, may differ from the minimal margin analyzed as a predictor for time-to-LTP, measured on the 4–8-week post-ablation CT as per reporting standards for ablation [1]. Another limitation of a post-ablation biopsy is the lack of immediate assessment that can guide additional ablation decisions intraprocedurally. To address these issues, intraprocedural 3D biopsy guidance and post-ablation tissue evaluation with real-time morphological and viability surrogates are implemented in an NIH-funded clinical trial (NCT01494324) currently enrolling. In addition, this trial focuses on correlating tissue findings with metabolic imaging tumor characteristics, 3D assessments of the ablation zone and genomics in an effort to develop disease and ablation-specific, predictive surrogate image biomarkers. Such a development may allow complete non-invasive assessment of the AZ in the future.

The only toxicity related to the post-ablation biopsy is the minimal risk of bleeding caused by the needle pass to obtain samples from the AZ. In our cohort, this risk was 0.6%, which is in accordance with rates reported in the literature [54,55,56].

The benefit of liver-directed locoregional therapies in OS has been demonstrated in randomized control trials (RCT) [5]. Ruers et al. reported 8-year survival of 35.9% vs. 8.9% in the combined therapy arm treated with RFA (±resection) in addition to chemotherapy vs. the group treated by chemotherapy alone [5]. It has also been shown that patients re-ablated for new metastases or LTP achieved longer OS than those that had LTP or new metastases that were not re-ablated [6,19]. The median OS in this cohort was 46 months since ablation and 84 months since the initial diagnosis of colorectal cancer. This is similar to the median survival of 45.6 months reported in prior RCT and within the range observed in other extensive retrospective studies with median OS ranging between 36 and 53.2 months after TA ablation [5,6,19]. The increased OS in the latter study might be explained by the fact that most patients were treated by ablation as the first liver-directed therapy, unlike this study where the majority of patients received TA for the treatment of post-hepatectomy recurrence [19]. Moreover, patients with increased CEA levels, prior thermal ablation and those who received adjuvant systemic chemotherapy after ablation had significantly worse survival compared to those treated by thermal ablation alone. The shorter survival in this subgroup of the cohort is the reflection of a more aggressive tumor biology requiring systemic therapy for multifocal progression of disease after liver tumor ablation. In this study, local tumor progression-free survival did not impact the overall survival, which could be explained by the local nature of the treatment, without accounting for other therapies that could contribute to patients’ overall survival. Longer follow-up and a larger cohort may allow a more meaningful analysis of factors impacting overall patient survival after CLM ablation.

Current oncologic guidelines support thermal ablation alone or in combination with surgery as long as all visible disease is eradicated [3,5]. The difference in OS between patients treated with TA and chemotherapy vs. chemotherapy alone, as well as the prolongation of survival for patients treated with TA for LTP and new tumors, support the value of complete tumor eradication by TA [5,19].

## 5. Conclusions

The present study indicates that a complete tumor ablation should include histopathological proof of complete tumor eradication in addition to the radiographic evidence of the targeted tumor ablation zone with wide margins. The incorporation of the histopathological assessment in the ablation clinical practice as a step to confirm complete tumor eradication could optimize TA as a local treatment for CLM, similar to the surgical standard.

## Figures and Tables

**Table 1:** Patient and tumor characteristics.

Patient (*n* = 107) and Tumor (*n* = 182) Characteristics	
Characteristic	Value
Age (y) *	59 (32–82)
Sex	
Female	42 (39)
Male	65 (61)
Race	
Asian/Far East/Indian Subcontinent	5 (5)
Black/African American	7 (7)
Other	1 (1)
Patient refused to answer	2 (2)
White	92 (92)
Tumor size (cm) **	2.0 (0.6–4.6)
LN status at staging of primary disease	
Positive	70 (65)
Negative	37 (35)
Synchronous CLM	77 (72)
Time between diagnosis of colorectal cancer and ablation (mo) *	31 (2–151)
No. of tumors treated per patient within protocol **	1.7 (1–9)

Unless otherwise indicated, data represent the number of patients, and data in parentheses are percentages. * Data are median values, and data in parentheses represent the range. ** Data are mean values, and data in parentheses represent the range. CLM = colorectal liver metastases.

**Table 2:** Tumor characteristics as predictors of local tumor progression (LTP).

Tumor Characteristics as Predictors of LTP (*n* = 178)			
	No. of Tumors	LTP Rate (%)	*p* Value
Biopsy			<0.001
Positive- Viable tumor	64	52 (33/64)	
Negative- Coagulation Necrosis	114	23 (25/114)	
Ablation Margin (mm)			<0.001
<5	33	70 (23/33)	
≥5	145	24 (35/145)	
Tumor size (cm)			0.036
≥3	26	50 (13/26)	
<3	152	30 (45/152)	
Ablation Modality			0.2
MWA	90	36 (32/90)	
RFA	88	30 (26/88)	
PET Guidance			0.3
Yes	131	31 (41/131)	
No	47	36 (17/47)	
CEA level (ng/mL [μg/L])			0.4
≤30	156	31 (50/156)	
>30	22	36 (8/22)	
EHD			0.4
Yes	86	31 (27/86)	
No	92	34 (31/92)	
Prior Liver Resection			0.034
Yes	147	29 (43/147)	
No	31	48 (15/31)	
Prior Systemic Chemotherapy			0.5
Yes	166	33 (54/166)	
No	12	33 (4/12)	
Prior HAIC			0.4
Yes	99	31 (31/99)	
No	79	34 (27/79)	
Post-Ablation Systemic Chemotherapy			0.1
Yes	140	35 (49/140)	
No	38	24 (9/38)	
Post-Ablation HAIC			0.3
Yes	60	35 (21/60)	
No	118	31 (37/118)	

LTP = local tumor progression; MWA = microwave ablation; RFA = radiofrequency ablation; PET = positron emission tomography; CEA = carcinoembryonic antigen; HAIC = hepatic artery-infusion chemotherapy.

**Table 3:** Univariate and multivariate analyses of factors associated with local tumor progression by using the competing-risks regression model.

Univariate and Multivariate Analyses of Factors Associated with Local Tumor Progression by Using the Competing-Risks Regression Model				
	Univariate Analysis	Multivariate Analysis		
Variable	*p* Value	Hazard Ratio	95% Confidence Interval	*p* Value
Biopsy result (V vs. N)	<0.001	2.4	1.4, 4.1	0.002
Minimal margin size < 5 mm	<0.001	3.5	2.0, 6.2	<0.001
Tumor size ≥3 cm	0.036	1.5	0.9, 2.7	0.133

V = viable; N = necrotic.

**Table 4:** Patient characteristics as predictors of overall survival.

Patient Characteristics as Predictors of OS (*n* = 106)			
	No. of Patients	Median OS (mo)	*p* Value
LTP			0.1
Yes	38	37	
No	68	49	
CEA level (ng/mL [μg/L])			<0.001
≤30	92	52	
>30	14	22	
EHD			0.1
Yes	54	42	
No	52	49	
Prior Liver Resection			0.1
Yes	83	49	
No	23	34	
Prior Systemic Chemotherapy			-
Yes	105	46	
No	1	-	
Prior HAIC			0.3
Yes	67	49	
No	39	36	
Post-ablation Systemic Chemotherapy			<0.001
Yes	78	36	
No	28	76	
Post-Ablation HAIC			0.9
Yes	33	49	
No	73	44	

OS = overall survival; LTP = local tumor progression; CEA = carcinoembrionic antigen; EHD = extrahepatic disease; HAIC = hepatic artery-infusion chemotherapy.

## Data Availability

The data that support the findings of this study are available from the corresponding author, (C.T.S.), upon reasonable request.

## Supplementary Materials

The following supporting information can be downloaded at: https://www.mdpi.com/article/10.3390/cancers14030693/s1, Table S1: Various ablation systems used in the study.
