# Supplementary material for: Biopsy and Margins Optimize Outcomes after Thermal Ablation of Colorectal Liver Metastases
Source: Cancers (Basel). 2022 Jan 29;14(3):693. doi: 10.3390/cancers14030693 (PMC8833800; doi:10.3390/cancers14030693)
Supplement: Supplementary file 1 [file cancers-14-00693-s001.zip › cancers-1472948-supplementary.pdf]

Table S1. Ablation systems used in the study.

Ablation modality	
**RFA**	**90/182 (49)**
Valleylab Cool-tip (Covidien, Mansfield, MA)	49/90 (54)
RITA StarBurst XLi (AngioDynamics, Latham, NY)	34/90 (38)
LeVeen (Boston Scientific, Marlborough, MA)	5/90 (6)
StarBurst Talon (AngioDynamics, Latham,NY)	2/90 (2)
**MWA**	**92/182 (51)**
Neuwave (NeuWave Medical, Madison, WI)	56/92 (61)
Emprint (Medtronic, Minneapolis, MI)	19/92 (21)
Amica (HS Hospital service S.P.A, Roma, Italy)	7/92 (8)
Microsulis (AngioDynamics, Latham, NY)	7/92 (8)
Acculis (AngioDynamics, Latham, NY)	2/92 (2)
Sota (Sota Medical, Cedar Grove, NJ)	1/92 (1)

Data represent the number of tumors treated and data in parentheses are percentages
